# How Cannabis Advertising and Price-Based Sales Promotion Shape Early Use: A National Study of Adolescents and Young Adults

**DOI:** 10.1080/10826084.2026.2670629

**Published:** 2026-05-18

**Authors:** Lei Xu, Shiqi Zhang, Ce Shang

**Affiliations:** a Center for Tobacco Research, The Ohio State University Comprehensive Cancer Center, The Ohio State University, Columbus, Ohio, USA; b Department of Internal Medicine, College of Medicine, Division of Medical Oncology, The Ohio State University Wexner Medical Center, Columbus, Ohio, USA

**Keywords:** Cannabis initiation, Adolescents and Young Adults (AYAs), Advertisement exposure, Price-based sales promotion, Public health policy

## Abstract

**Purpose:**

To examine associations between cannabis advertising and price-based promotions and cannabis initiation among U.S. adolescents and young adults (AYAs).

**Methods:**

We used two-wave panel data (2024–2025) from a nationally representative sample of U.S. AYAs aged 15–20 who were current cannabis users or susceptible to cannabis use. Initiation was defined as: recent use initiation (past 30-day use in wave 2, no past-30-day use in wave 1) and lifetime initiation (ever use in wave 2, never use in wave 1). Advertising and price-based promotion exposure were measured over the past six months. Logistic and split-population survival models were estimated to examine associations with initiation.

**Results:**

Exposure to any advertising was positively associated with initiation (logistic: *β* = 2.25, *p* < 0.05; split-population: *β* = 1.90, *p* < 0.05). When exposure was measured by number of advertising channels, exposure through one channel (logistic: *β* = 1.73; split-population: *β* = 1.42; both *p* < 0.01) and three or more channels (logistic: *β* = 2.02; split-population: *β* = 1.73; both *p* < 0.05) were significantly associated with higher initiation risk. Price-based promotion exposure was not significantly associated with recent initiation but was positively associated with lifetime initiation (logistic: *β* = 1.09, *p* < 0.05; split-population: *β* = 0.84, *p* < 0.05).

**Conclusions:**

Cannabis advertising and price-based promotions are linked to initiation among AYAs. As cannabis commercialization expands amid limited marketing oversight, these findings highlight the need for policies that limit AYAs’ exposure to cannabis advertising and price promotion.

## IMPLICATIONS AND CONTRIBUTION SUMMARY

This study provides one of the first national evidence linking cannabis advertising exposure and price-based sales promotions to initiation among adolescents and young adults (AYAs). Findings highlight how advertisement strategies shape AYA use and inform regulatory efforts to reduce early exposure and support prevention strategies targeting commercial determinants of AYA cannabis behavior.

## Introduction

Cannabis use among adolescents and young adults (AYAs) in the USA has become an increasingly urgent public health concern, particularly as cannabis legalization expands across states (Degenhardt et al., 2016). As of May 2025, recreational cannabis use is legal in 24 U.S. states, the District of Columbia, and three U.S. territories, while medical use is permitted in 47 states and additional jurisdictions (Baldwin et al., 2024; Center for Disease Control & Prevention, 2024; Hammond et al., 2021; Mr Cannabis Law, 2025). Several other states are actively considering legalization to legalize recreational cannabis, such as Texas (Norton, 2025). In parallel with legalization trends, cannabis use among AYAs remains widespread. In 2024, 25.8% of 12th-grade students reported past-year cannabis use, and 5.1% indicated daily use over the previous 30 days (Miech et al., 2024). Among young adults aged 18–25, cannabis use prevalence is higher, with approximately one-third reporting past-year use, according to national survey estimates (Samhsa, 2023).

The normalization of cannabis use, and the rapid commercialization of the cannabis industry have introduced new environmental risk factors, including advertisements and price-based sales promotion, that may influence initiation and sustained use among AYA populations (D’Amico et al., 2015; Dai, 2017; Trangenstein et al., 2021). Unlike the tobacco industry, where federal restrictions have significantly curtailed youth-directed marketing, cannabis advertising remains governed under a more fragmented regulatory landscape, often allowing a wide array of advertising formats, including social media and digital platforms that can reach underage audiences (Krauss et al., 2017; Whitehill et al., 2020). While retail environments often restrict youth access, youth exposure more commonly occurs through social media platforms (e.g., Instagram, TikTok, Snapchat) and broader digital channels such as websites, streaming services, and online advertisements. Early evidence suggests that such marketing tactics may reach underage audiences despite regulatory restrictions (D’Amico et al., 2018; Fiala et al., 2020; Miech et al., 2022), leading to the normalization of cannabis and potentially influencing early use decisions.

This study is the first to provide nationally representative estimates of cannabis advertising exposure across multiple source types, with analysis of sociodemographic, behavioral, and policy-related correlates among U.S. AYAs. Previous research, largely focused on descriptive patterns, has demonstrated that AYA exposure to cannabis advertising is associated with more favorable attitudes toward cannabis, increased potential to use, and higher rates of reported use (Barry & Glantz, 2016; D’Amico et al., 2015; Dai, 2017; Firth et al., 2022; Hust et al., 2020; Johnston et al., 2020; Kelleghan et al., 2020; Noël et al., 2024; Rup et al., 2020; Trangenstein et al., 2019, 2021; Whitehill et al., 2020; Willoughby et al., 2024; Yang et al., 2024). In particular, studies have linked price-based sales promotion and multi-channel advertising exposure to a higher likelihood of cannabis experimentation among adolescents (D’Amico et al., 2015; Trangenstein et al., 2019, 2021; Whitehill et al., 2020). However, the existing literature has largely been descriptive, cross-sectional, and has focused primarily on isolated exposure effects on current cannabis use prevalence, without adequately capturing how these exposures contribute to cannabis use initiation (Jackson et al., 2018; Trangenstein et al., 2021; Whitehill et al., 2020).

Furthermore, few studies have systematically examined how exposure may be linked to cannabis initiation, the first-time transition from nonuse to use, in a U.S. nationally representative and longitudinal sample. Prior work has typically focused on general cannabis use or frequency (Dai, 2017; Trangenstein et al., 2022), or use localized sample such as students from a small number of schools (Kelleghan et al., 2020). Moreover, while some international studies have examined advertising exposure and youth intentions to use, few have directly tested how these exposures shape cannabis initiation in the U.S. context, particularly among AYAs navigating an increasingly commercialized market (Noël et al., 2024).

This study addresses these critical gaps by using two-wave panel data from a nationally representative survey of U.S. AYAs aged 15–20, conducted in 2024 and 2025. We focus explicitly on cannabis initiation and examine how exposure to cannabis advertising and price-based sales promotion are associated with initiation. Our analytic approach is based on survival analysis and contains robustness checks using state-level cannabis advertising exposure and price-based sales promotion data. By linking advertising environments with individual behavioral pathways, this study offers new insights into how cannabis advertising may influence early cannabis initiation. To our knowledge, this is the first longitudinal study to estimate the role of cannabis advertising and promotion in shaping cannabis initiation among U.S. AYAs, with implications for both state-level policy and public health interventions.

## Methods

### Study sample

Data were collected through the Ipsos Knowledge Panel, a probability-based online panel designed to be representative of the U.S. population. Panel members are recruited using address-based sampling (ABS) from the U.S. Postal Service Delivery Sequence File, ensuring coverage of households. The Knowledge Panel is designed to be nationally representative of the U.S. population at recruitment.

In June 2024 and January 2025, we conducted two waves of nationally representative longitudinal surveys of U.S. AYAs (ages 15–20) who met the following criteria: (1) currently use cannabis or (2) are susceptible to future cannabis use (Barrington-Trimis et al., 2020). Individuals who neither used cannabis nor met susceptibility criteria were excluded. These surveys collected detailed information on cannabis use behaviors, advertising exposure, price-based sales promotion, substance use behaviors, and sociodemographic characteristics.

The richness of the data allows for detailed analysis of the relationship between advertising exposure and cannabis initiation among AYAs. For this study, since we focus on the subsample of 532 respondents who participated in both wave 1 and wave 2, 964 responses from those who only participated in one wave are dropped. To assess potential attrition bias, Supplementary Appendix Tables A.1 and A.2 compare respondents who completed both survey waves with those who completed only a single wave, including a focused comparison with Wave-1 dropouts. Across these analyses, most demographic and behavioral characteristics do not differ significantly, suggesting minimal selection bias and supporting the validity of restricting the analytical sample to respondents participating in both waves. One exception is cannabis use purpose, which differs significantly between groups, with respondents retained in both waves more likely to report recreational use. The difference may reflect differential engagement across user types. However, all other variables are well balanced, and cannabis use purpose is controlled for in the regression models, this difference is unlikely to meaningfully affect the main findings.

To examine cannabis initiation, we defined two distinct outcomes reflecting different stages of cannabis use onset. Recent cannabis initiation is defined as the transition from no cannabis use in the past 30 days at wave 1 to any use within the last 30 days at wave 2 and does not necessarily imply first-ever cannabis use. We restrict our analytic sample to those who reported no cannabis use within 30 days at Wave 1. This outcome captures the onset of current cannabis use between waves and may include individuals who had previously experimented with cannabis prior to Wave 1. The subsample includes 31 respondents who report cannabis use within the last 30 days in wave 2 but no cannabis uses within the last 30 days in wave 1, and 107 respondents who report no cannabis use within the last 30 days for both wave 1 and 2.

Lifetime cannabis initiation captures the transition from never having used cannabis at Wave 1 to ever using cannabis by Wave 2. For this outcome, we restrict our sample to those who reported never use cannabis at Wave 1. This subsample contains 242 respondents, including 46 new initiators (no use at wave 1, use at wave 2) and 196 consistent nonusers (no use reported at either wave), see detailed statistics in Table 1. Table 1 also includes the key variables, such as advertising exposure and price-based sales promotion, as well as individual and household characteristics collected at baseline.

### Human subjects and ethical approval

This study was reviewed and determined exempt by The Ohio State University Institutional Review Board (IRB), as it involved minimal risk and used survey procedures without collecting identifiable private information (IRB protocol #2022B0032). All study procedures were conducted in accordance with the principles of the Helsinki Declaration.

### Variables

#### Outcome variables

##### Lifetime cannabis initiation indicator.

The binary variable captures new lifetime initiation of cannabis use between survey waves. The variable is derived from the survey question, “Have you ever used cannabis products, even one or two times?” The indicator is coded as 1 if the respondent reported never having used cannabis in wave 1 but reported ever having used cannabis in Wave 2. The indicator is coded as 0 if individuals reported no ever use in two waves.

##### Recent use initiation indicator.

This binary variable captures the onset of recent (past–30-day) cannabis use between survey waves. The variable is based on the survey question, “In the past 30 days, have you used cannabis products, even one or two times?” The indicator is coded as 1 if the respondent reported cannabis use in the past 30 days during Wave 2, but having reported no past-30-day-use in Wave 1. The indicator is coded as 0 otherwise, including individuals who did not use in both waves. This definition captures the onset of recent (past-30-day) use between waves and may include individuals with prior lifetime experimentation. We therefore interpret this outcome as initiation of recent use rather than first-ever use.

#### Explanatory variables

##### Cannabis advertising exposure (individual-level).

Exposure to cannabis advertising was measured using a self-reported survey question: “In the last 6 months, have you seen cannabis being advertised or promoted in any of the following places? You can choose more than one.” Response options included postal mail; websites; email or text messages; retail and event-based locations (e.g., bars, festivals, kiosks, inside/outside cannabis stores); television or radio; billboards, posters, flyers, and print media; movie theaters; public transit; social media platforms; community websites or apps; and messaging applications.

From this question, we constructed two measures: (1) a binary indicator for any advertising exposure (1 = exposed to at least one channel; 0 = no exposure), and (2) a categorical variable capturing the number of distinct advertising channels (0, 1, 2, ≥3), top-coded to limit sparsity at higher values.

##### Price-based sales promotions (individual-level).

Exposure to price-based sales promotions was measured using a binary indicator capturing whether the respondent noticed any cannabis-related discounts or sales in the past 6 months (1 = any price-based sales promotion exposure; 0 = no exposure). This measure is based on the question, “In the past 6 months, have you noticed the following cannabis discounts or sales? You can choose more than one.” The options contain quantity discount (e.g., BOGO), coupons, direct price discount (e.g., 20% off), free shipping, free gifts with purchase, and other. These items represent economic incentives rather than advertising content and are analytically distinct from the advertising exposure measure. Although respondents could select multiple promotion types, the analytic variable was dichotomized to indicate any *versus* no exposure due to the skewed distribution of promotion counts.

##### Substance use behavior and context variables (individual-level).

Behavioral covariates included cannabis use purpose (medical use, neither recreational nor medical, both, recreational use as reference) and the number of other substances used^1^ (e.g., tobacco). These variables were included in baseline models to adjust for individual predispositions that may confound the association between advertising exposure and cannabis initiation.

##### Demographics variables (individual-level).

Control variables include age (15–20); gender (female, male as reference); ethnicity (Hispanic, non-Hispanic as reference); education (some college or associate’s degree, bachelor’s degree or higher, high school graduate or no high school diploma as reference)^2^; household size; living regions (Midwest, West, South, Northeast as a reference). Educational attainment is widely used as an indicator of socioeconomic status in public health research, including among AYAs (Braveman et al., 2005; Patrick et al., 2012).

##### State cannabis legalization status (2025).

State cannabis legalization status was obtained from the National Conference of State Legislatures’ compilation of medical and recreational cannabis laws in 2025. States were categorized as fully legal (both medical and recreational cannabis legal), mixed (medical cannabis legal or limited access programs such as CBD/low-THC only), or fully illegal (no legal cannabis access). This external policy measure was linked to respondents based on their state of residence during the survey period.

#### Analysis method

The primary objective of this study is to assess how exposure to cannabis advertising and price-based sales promotion is associated with cannabis initiation among U.S. AYAs. To model the likelihood of cannabis initiation, we first estimate a simple survival model using logistic regression. First, we applied logistic regression to model the probability of initiating cannabis use ($initiation_{i}$) as a function of cannabis advertising exposure, price-based sales promotion, and control variables ($X_{i}$) for both recent use initiation and lifetime initiation (Equation 1). Separate models were estimated for recent cannabis initiation and lifetime cannabis initiation.

(1) $$\mathrm{logit}\left[P({\text{initiation}}_{i}=1)\right]=\alpha_{0}+\alpha_{1}MarExp_{i}+\alpha_{2}Promo_{i}+X_{i}^{\prime }\gamma$$

where $MarExp_{i}$: any exposure to cannabis advertisements

$Promo_{i}$: any exposure to price-based sales promotions

$X_{i}$: vector of demographic and socioeconomic controls (e.g., age, gender, education, etc.)

To account for the fact that a subset of AYAs may never initiate cannabis use during the study period, we further estimated a split-population survival model with cloglog distributions (Kostova et al., 2015). The survival model estimates the likelihood that an individual ever initiates cannabis use as a function of marijuana advertising exposure, price-based sales promotion exposure, and demographic and contextual covariates. This modeling framework allows advertising and promotion exposures to influence the likelihood of initiation, while explicitly accounting for a subpopulation of adolescents who do not initiate cannabis use during the observation period. For all models, we report estimated coefficients for all predictors.

## Results

Table 1 summarizes major characteristics for two analytic subsamples, one for lifetime initiation (*N* = 242) and one for recent use initiation (*N* = 138). In the lifetime initiation group, 19% initiated cannabis use between waves, while 81% remained never-users. In the recent use group, 22.5% initiated recent use, and 77.5% remained non-recent users. Across both groups, most respondents reported some exposure to cannabis advertising, with nearly 70–80% seeing ads in at least one channel. Price-based sales promotion exposure was noted by 11–21% of respondents. Motivation for cannabis use varied, with recreational use or combined with medical reasons being the most reported. Detailed comparisons between initiators and non-initiators are presented in Supplementary Appendix Tables A.6 and A.7.

Table 2 presents coefficients from both logistic and split population survival models examining the associations between cannabis advertising exposure, price-based sales promotion, and a range of individual-level covariates with cannabis recent use initiation among U.S. AYAs. The logistic model estimates the overall probability of initiation, whereas the split-population model accounts for a subgroup of individuals who are not at risk of initiating (“never initiators”), such that estimated effects reflect the likelihood of initiation among those at risk. The results show that exposure to any cannabis advertisement significantly increases the probability of recent use initiation (logistic model: *β*= 2.25, *p* < 0.05; split-population model: *β* = 1.90, *p* < 0.05). In models (2) and (4), the analysis shifts from a binary indicator of any advertisement exposure to a categorical measure capturing the number of exposure channels. Exposure through one channel is associated with an increase in initiation probability (logistic model: *β* = 1.73, *p* < 0.01; split-population model: *β*= 1.42, *p* < 0.01). Exposure through three or more channels is associated with the larger probability of initiation (logistic: *β* = 2.02, *p* < 0.05; split-population model: *β*= 1.73, *p* < 0.05).

Across covariates, age shows the strongest association with recent cannabis initiation. Relative to age 20, adolescents aged 15 have a substantially higher probability of initiation (*β* = 2.68–3.37, *p* < 0.05 or 0.01). Estimates for ages 16 and 17 are positive but not statistically significant, while coefficients for ages 18 and 19 are smaller and still not significant. Having some college education is associated with a higher likelihood of cannabis initiation in one survival specification (*β*= 1.75, *p* < 0.05) relative to individuals with a high school diploma or less, whereas holding a bachelor’s degree or higher is not significantly associated with initiation. In addition, cannabis use purpose (medical, both medical and recreational, or neither) is not significantly associated with recent initiation.

Table 3 presents result for lifetime cannabis initiation using both logistic and split population survival models. Across both the logistic and split-population survival specifications, exposure to cannabis advertising shows no statistically significant association with lifetime cannabis initiation. Exposure to price-based sales promotions is associated with a higher probability of lifetime cannabis initiation across both modeling frameworks. Exposure to price-based promotions is associated with a higher probability of lifetime initiation (logistic model: *β*= 1.09, *p* < 0.05; split-population model: *β*= 0.84, *p* < 0.05). In contrast, exposure to cannabis advertising and the number of advertising exposure channels are not associated with significant changes in the probability of lifetime initiation.

Hispanic respondents show a higher probability of lifetime initiation than non-Hispanic respondents, with coefficients of approximately 1.0–1.3 across models (*p* < 0.05). Educational attainment shows a strong effect. Holding a bachelor’s degree or higher is associated with an increase in the probability of lifetime initiation across both logistic and survival specifications (*β*= 1.9–2.2, *p* < 0.01), relative to individuals with a high school diploma or less, whereas some college education is not significantly associated with initiation. Medical cannabis use is associated with the larger initiation probability, with coefficients ranging from approximately 1.5–1.8 relative to recreational use across all models (*p*< 0.01 or *p* < 0.001). Regional differences are also observed, with residence in the West associated with a lower probability of lifetime initiation compared with the Northeast (*β*= −2.0 to −2.3, *p* < 0.05). Finally, medical-only cannabis legality is associated with a lower probability of lifetime initiation relative to fully illegal states (*β*= 1.5–1.8, *p* < 0.01 or *p* < 0.001).

We constructed state-level exposure measures by aggregating weighted individual responses and re-estimated the models (Supplementary Appendix Table A.3). The state-level exposure variables were not significantly associated with recent initiation in either the logistic or split-population specifications. We estimated models including only significant variables, excluding insignificant covariates to increase degrees of freedom (Supplementary Appendix Tables A.4 and A.5). The results remain substantively consistent with the fully adjusted models. In addition, VIF diagnostics suggest that multicollinearity is not a concern in our specifications. The mean VIF equals 1.79 for the recent use models and 2.20 for the lifetime use models, and all individual VIF values fall comfortably below conventional cutoffs (O’brien, 2007).

## Discussions

This study is the first to provide novel evidence on the association of cannabis advertisement exposure and cannabis use initiation among U.S. AYAs, using a nationally representative survey. Focusing explicitly on initiation, we distinguish between recent use initiation (transition into current use) and lifetime initiation (first-ever use). Our findings show that individual-level exposure to cannabis advertising is associated with recent cannabis initiation, particularly when exposure occurs across multiple channels. This pattern is consistent with a non-linear relationship between advertising exposure and initiation, where initial exposure (one channel) may increase awareness, and more intensive exposure (three or more channels) may reinforce behavioral influence. In contrast, exposure through two channels may not represent a distinct or sufficiently strong level of engagement. In contrast, price-based sales promotions are more strongly associated with lifetime initiation. Our primary contribution is capturing how commercial determinants, such as advertisement and price-based sales promotion, interact with young-level predispositions to shape early-stage cannabis use.

Our findings move beyond existing descriptive studies by offering a first nationally representative analysis of multi-channel cannabis advertising exposure and cannabis use initiation among AYAs. This study demonstrates that exposure is not only widespread but also systematically linked to cannabis use behaviors such as recent initiation, suggesting that commercial cannabis advertising influences beyond simple brand recognition or product awareness (Rup et al., 2020; Trangenstein et al., 2019; Whitehill et al., 2020). Furthermore, this study reflects cannabis advertising strategies may act as behavioral triggers for use initiation or transition. This finding aligns with marketing theories that consider advertising as not only informative but also transformative in shaping preferences and risk perceptions (Bonfrer et al., 2020; Morwitz et al., 1993). Our study provides stronger evidence that cannabis advertising functions not only as an informational tool but also as a socializing force that normalizes use, especially in the context of permissive and under-regulated cannabis markets (see Supplementary Appendix I and Table A.3).

Unlike previous literature that primarily examines cannabis current use prevalence or frequency or medical cannabis use (Barry & Glantz, 2016; Cui et al., 2025; D’Amico et al., 2015, 2018; Dai, 2017; DiGuiseppi et al., 2023; Firth et al., 2022; Hust et al., 2020; Jackson et al., 2018; Johnston et al., 2020; Kelleghan et al., 2020; Noël et al., 2024; Rup et al., 2020; Trangenstein et al., 2019, 2022; Whitehill et al., 2020; Willoughby et al., 2024; Yang et al., 2024), this study uniquely focuses on cannabis use initiation, capturing the translational moment from nonuse to first-time use by using a longitudinal nationally representative sample. Some prior work has often emphasized risk factors, such as concurrent use of alcohol and tobacco, as key predictors of cannabis use (DiGuiseppi et al., 2023; Kelleghan et al., 2020). However, our models show no significant relationship between other substance use and cannabis initiation, suggesting that advertisement-related factors may exert a stronger influence on early cannabis use than prior use of other substances. Furthermore, while earlier research has found strong geographic effects tied to legal status, our analysis reveals less consistent patterns across states once individual- and state-level exposure variables are included (Goodman et al., 2020; Midgette & Reuter, 2020). These differences may be due to evolving marketing strategies, regulatory patchworks, or shifting cultural perceptions of cannabis among AYAs. Our results emphasize the need to reassess traditional predictors of initiation in light of an increasingly commercialized cannabis environment.

From a policy standpoint, these findings highlight the need for proactive safeguards in emerging cannabis markets, which has also been emphasized by previous studies calling for restrictions on youth-targeted advertising, price-based sales promotion, and digital marketing (Cui et al., 2025; Dai, 2017; DiGuiseppi et al., 2023; Firth et al., 2022; Hust et al., 2020; Jackson et al., 2018; Noël et al., 2021, 2024; Trangenstein et al., 2022; Whitehill et al., 2020). Although our findings show that cannabis advertising exposure is associated with current use initiation among AYAs, the current U.S. regulatory framework remains fragmented and insufficiently protective. At the federal level, oversight is led by agencies such as the Federal Trade Commission (FTC), which prohibits deceptive or unsubstantiated marketing claims, and the Food and Drug Administration (FDA), which bans unauthorized health claims about cannabis products (FDA, 2024; Nagle, 2019). However, because cannabis remains a Schedule I controlled substance, federal enforcement is limited, and most advertising regulations are state-specific (Bodie et al., 2022; Amy et al., 2022).

States with legalized cannabis markets have adopted various advertising restrictions to protect minors, including content bans, proximity rules, and age-gating for digital platforms (MediaJel, 2025). For instance, regulatory bodies such as the California Department of Cannabis Control (DCC) and the Colorado Marijuana Enforcement Division (MED) have established and oversee regulations on advertising channels and content aimed at youth (Hinckley et al., 2024; McElfresh, 2024). Despite these measures, our results indicate that exposure to cannabis marketing remains common and consequential, even among adolescents who had not previously used cannabis. This finding raises questions about the effectiveness and enforcement of existing policies, particularly in digital media where age verification and content monitoring are inconsistent (Trangenstein et al., 2019; Whitehill et al., 2020).

States and local communities should consider restrictions on youth-oriented advertising, including limits on discounting strategies, social media targeting, and cross-platform exposure. Drawing from lessons learned in tobacco control, comprehensive marketing restrictions, coupled with strong enforcement mechanisms, could help mitigate early exposure and reduce cannabis initiation among AYAs (Barry & Glantz, 2016; National Cancer Institute & CDC, 2016). Furthermore, these insights suggest that prevention strategies should move beyond individual-level messaging to also target environmental and structural drivers of substance use, including commercial determinants.

Despite the strengths of a nationally representative design and extensive robustness checks, this study has several limitations. First, the exposure measures rely on self-report, which introduces potential recall or social desirability bias, especially for advertising viewed in less memorable formats such as digital banners or short videos. Second, our two-wave design only captures initiation within a 6-month window, thus longer-term tracking is needed to understand the sustained impact of advertising on cannabis trajectories. Third, although initiation is defined using information from Waves 1 and 2, our regression framework does not jointly model outcomes across waves, preventing a direct comparison of Wave 1 nonusers with Wave 2 recent users. Finally, limited within-person variation in advertising and promotion exposure between waves restricts our ability to estimate change-based models. Because relatively few respondents experienced meaningful shifts in exposure, change specifications lack sufficient statistical power. Future research with longer follow-up periods or greater exposure variability may better capture dynamic exposure effects.

In conclusion, this study highlights the critical role that cannabis advertising exposure plays in shaping early use patterns among AYAs. As cannabis legalization and commercialization continue to expand, our findings emphasize the need for policy attention to marketing practices that disproportionately reach young people. Future research should leverage experimental or quasi-experimental designs, such as natural experiments from policy shifts, to better establish causal links between advertising and initiation. A deeper understanding of these mechanisms will be essential to designing effective regulatory strategies and public health interventions in the years ahead. To protect AYAs during this critical stage of development, regulatory efforts must go beyond limiting product access and directly address the pervasive advertisement strategies that shape AYA perceptions, preferences, and initiation behaviors.

## Supplementary Material

Appendix

Supplemental data for this article can be accessed online at https://doi.org/10.1080/10826084.2026.2670629.

## Figures and Tables

**Table 1.** Descriptive statistics of U.S. AYAs participating in both survey waves (June 2024 and January 2025).

Variables	Observations (percent)	
	Lifetime initiation	Recent use initiation
Cannabis use, advertisement, and promotion exposure:		
Cannabis use		
Initiation	46 (19.01%)	31 (22.46%)
No use	196 (80.99%)	107 (77.54%)
Exposed to any cannabis advertisement		
Yes	166 (68.60%)	109 (78.99%)
No	76 (31.40%)	29 (21.01%)
Number of cannabis advertisement channels exposed (past 6 months)		
No exposure	76 (31.40%)	29 (21.01%)
1	85 (35.12%)	42 (30.43%)
2	27 (11.16%)	25 (18.12%)
≥3	54 (22.31%)	42 (30.44%)
Exposed to any cannabis price-based sales promotion (past 6 months)		
Yes	28 (11.57%)	29 (21.01%)
No	211 (87.19%)	109 (78.99%)
Don’t know	3 (1.24%)	–
Demographics		
Age		
15	62 (25.62%)	14 (10.14%)
16	62 (25.62%)	22 (15.94%)
17	31 (12.81%)	10 (7.25%)
18	38 (15.70%)	21 (15.22%)
19	25 (10.33%)	37 (26.81%)
20	24 (9.92%)	24 (24.64%)
Gender		
Female	115 (47.52%)	62 (44.93%)
Male	124 (51.24%)	76 (55.07%)
Missing	3 (1.24%)	
Ethnicity		
Hispanic	49 (20.25%)	23 (16.67%)
Non-Hispanic	193 (79.75%)	115 (83.33%)
Education		
High school or below	172 (71.07%)	44 (31.88%)
Some college	61 (25.21%)	49 (35.51%)
Bachelor’s degree or higher	9 (3.72%)	45 (32.61%)
Substance use behavior and context		
Cannabis use purpose		
Recreational	18 (7.44%)	15 (10.87%)
Medical	80 (33.06%)	73 (52.90%)
Both	46 (19.01%)	22 (15.94%)
Neither medical nor recreational	98 (40.49%)	28 (20.29%)
Region		
Northeast	41 (16.94%)	28 (20.29%)
Midwest	62 (25.62%)	36 (26.09%)
South	95 (39.26%)	52 (37.68%)
West	44 (18.18%)	22 (15.94%)
Legality		
Fully legal	113 (46.69%)	63 (45.65%)
Fully illegal	21 (8.68%)	13 (9.42%)
Mixed	108 (44.63%)	62 (44.93%)
	Mean (SD)	
Household size	3.84 (1.39)	3.92 (1.26)
Number of other substances	0.33 (0.67)	0.54 (0.73)
**Observations**	**242**	**138**

The number of other substances has a range of 0–4.

**Table 2.** Logistic and split population survival model estimates of cannabis use initiation, recent use initiation (*N* = 138).

	Dependent variable: cannabis initiation (recent use)			
	Logit model		Split population model	
	(1)	(2)	(3)	(4)
Advertisement and promotion exposure				
Exposed to any cannabis advertisement	2.254* (0.921)		1.895* (0.814)	
Number of cannabis advertisement channels exposed (ref: 0)				
1		1.731** (0.617)		1.419** (0.504)
2		0.399 (0.786)		0.284 (0.684)
≥3		2.015* (0.973)		1.726* (0.803)
Exposed to any cannabis price-based sales promotion	0.428 (0.561)	1.017 (0.573)	0.325 (0.451)	0.893 (0.462)
Demographics				
Age (ref: 20)				
15	3.260* (1.272)	3.372* (1.316)	2.681** (0.974)	2.957** (1.084)
16	1.153 (1.154)	1.742 (1.174)	0.923 (0.886)	1.484 (0.988)
17	1.042 (1.302)	1.723 (1.334)	0.901 (1.045)	1.320 (1.052)
18	−0.636 (0.958)	−0.174 (0.983)	−0.504 (0.835)	−0.130 (0.851)
19	0.564 (0.746)	0.813 (0.777)	0.583 (0.632)	0.823 (0.668)
Gender (ref: male)				
Female	−0.286 (0.503)	−0.329 (0.517)	−0.255 (0.425)	−0.246 (0.434)
Ethnicity (ref: non-Hispanic)				
Hispanic	0.103 (0.670)	0.354 (0.657)	0.156 (0.534)	0.334 (0.534)
Education (ref: high school or below)				
Some college	1.719 (1.026)	1.942 (1.046)	1.432 (0.771)	1.749* (0.865)
Bachelor’s degree or higher	0.121 (1.167)	0.410 (1.193)	0.108 (0.897)	0.368 (0.979)
Household size	−0.171 (0.218)	−0.239 (0.223)	−0.120 (0.223)	−0.187 (0.189)
Substance use behavior and context				
Cannabis use purpose (ref: recreational)				
Neither medical nor recreational	1.264 (1.081)	1.428 (1.152)	1.107 (0.893)	1.282 (0.974)
Medical	1.267 (0.854)	1.527 (0.930)	1.036 (0.696)	1.288 (0.785)
Both	1.099 (0.974)	1.554 (1.046)	0.890 (0.785)	1.246 (0.867)
Number of other substances	−0.210 (0.393)	−0.161 (0.417)	−0.213 (0.319)	−0.113 (0.342)
Region (ref: northeast)				
Midwest	0.132 (0.727)	−0.065 0.739	−0.094 (0.596)	−0.309 (0.609)
South	0.454 (0.807)	0.665 (0.824)	0.273 (0.684)	0.443 (0.677)
West	0.827 (0.846)	0.765 (0.862)	0.659 (0.685)	0.523 (0.705)
Legality (ref: fully illegal)				
Fully legal	−0.114 (0.954)	0.293 (0.972)	−0.278 (0.972)	0.181 (0.838)
Mixed	−0.488 (0.838)	−0.618 (0.947)	−0.560 (0.761)	−0.512 (0.790)
Constant	−5.075* (2.204)	−4.865* (2.231)	−4.474* (1.738)	−4.508* (1.854)
Observations	138	138	138	138

Columns (1)–(2) present logistic regression coefficients (log-odds). Columns (3)–(4) present split-population survival model coefficients from complementary log-log specifications. Standard errors are reported in parentheses. Columns (1) and (3) include the binary indicator for any cannabis advertising exposure. Columns (2) and (4) include the categorical measure capturing the number of cannabis advertising channels.

* *p* < 0.05

** *p* < 0.01

**Table 3.** Logistic and split population survival model estimates of cannabis use initiation, lifetime use initiation (*N* = 242).

	Dependent variable: cannabis initiation (lifetime use)			
	Logit model		Split population model	
	(1)	(2)	(3)	(4)
Advertisement and promotion exposure				
Exposed to any cannabis advertisement	0.274 (0.460)		0.260 (0.386)	
Number of cannabis advertisement channels exposed (ref: 0)				
1		−0.279 (0.456)		−0.225 (0.378)
2		−0.029 (0.584)		−0.021 (0.494)
≥3		−0.841 (0.917)		−0.610 (0.806)
Exposed to any cannabis price-based sales promotion	0.969 (0.544)	1.088* (0.531)	0.717 (0.433)	0.843* (0.422)
Demographics				
Age (ref: 20)				
15	−1.628 (0.861)	−1.630 (0.855)	−1.444 (0.774)	−1.497 (0.775)
16	−0.920 (0.836)	−0.948 (0.831)	−0.783 (0.745)	−0.836 (0.753)
17	−0.804 (0.854)	−0.790 (0.858)	−0.717 (0.760)	−0.744 (0.771)
18	−0.270 (0.760)	−0.392 (0.765)	−0.268 (0.669)	−0.405 (0.684)
19	0.655 (0.828)	0.600 (0.829)	0.487 (0.709)	0.379 (0.720)
Gender (ref: male)				
Female	−0.363 (0.402)	−0.329 (0.403)	−0.355 (0.340)	−0.324 (0.343)
Ethnicity (ref: non-Hispanic)				
Hispanic	1.250* (0.528)	1.189* (0.525)	1.039* (0.437)	0.979* (0.436)
Education (ref: high school or below)				
Some college	0.117 (0.566)	0.174 (0.562)	0.107 (0.481)	0.133 (0.476)
Bachelor’s degree or higher	2.207** (0.817)	2.150** (0.827)	1.919** (0.640)	1.852** (0.643)
Household size	0.075 (0.151)	0.089 (0.152)	0.046 (0.130)	0.052 (0.131)
Substance use behavior and context				
Cannabis use purpose (ref: recreational)				
Neither medical nor recreational	0.315 (0.763)	0.245 (0.757)	0.434 (0.663)	0.347 (0.656)
Medical	1.758*** (0.517)	1.680** (0.514)	1.548*** (0.451)	1.454** (0.449)
Both	0.523 (0.660)	0.413 (0.673)	0.534 (0.583)	0.434 (0.600)
Number of other substances	−0.098 (0.286)	−0.025 (0.291)	−0.053 (0.230)	−0.001 (0.236)
Region (ref: northeast)				
Midwest	0.005 (0.611)	−0.087 (0.614)	−0.004 (0.523)	−0.101 (0.526)
South	−0.213 (0.599)	−0.280 (0.604)	−0.251 (0.524)	−0.321 (0.525)
West	−2.189* (0.856)	−2.282** (0.870)	−2.019** (0.726)	−2.090** (0.745)
Legality (ref: fully illegal)				
Fully legal	−0.659 (0.686)	−0.697 (0.687)	−0.530 (0.555)	−0.518 (0.546)
Mixed	−1.756* (0.688)	−1.792** (0.696)	−1.528** (0.559)	−1.513** (0.558)
Constant	−1.183 (1.099)	−0.758 (1.112)	−1.271 (0.947)	−0.854 (0.956)
Observations	242	242	242	242

Columns (1)–(2) present logistic regression coefficients (log-odds). Columns (3)–(4) present split-population survival model coefficients from complementary log-log specifications. Standard errors are reported in parentheses. Columns (1) and (3) include the binary indicator for any cannabis advertising exposure. Columns (2) and (4) include the categorical measure capturing the number of cannabis advertising channels.

* *p* < 0.05

** *p* < 0.01

*** *p* < 0.001.

## Footnotes

1 Respondents were shown the following product list and were allowed to select multiple options: cigarettes; e-cigarettes (e.g., JUUL, PUFF BAR); heated tobacco products (e.g., IQOS); cigarillos and little cigars (e.g., Swisher Sweets); oral nicotine pouches (e.g., ZYN); smokeless tobacco (e.g., chewing tobacco, snuff, or snus); hookah or pipe tobacco; alcohol; and an “other” category with write-in responses.

2 We assessed multicollinearity between age and education using VIFs, with all values below 4 (mean VIF = 2.38), indicating no concerned collinearity.
