# Supplementary material for: How Cannabis Advertising and Price-Based Sales Promotion Shape Early Use: A National Study of Adolescents and Young Adults
Source: Subst Use Misuse. Author manuscript; Available in PMC 2026 Oct 8. (PMC13644041; doi:10.1080/10826084.2026.2670629)
Supplement: Supplementary file 1 [file NIHMS2211176-supplement-Appendix.docx]

**Appendix**

**I. State-Level Exposure Measures and Robustness Analyses**

Cannabis Advertising Exposure (State-level)

To assess the robustness of individual-level exposure measures and reduce concerns about reverse causality, we constructed a state-level cannabis advertising exposure measure by aggregating individual responses within each state. Specifically, we calculated the weighted state-level mean of the binary indicator for any cannabis advertising exposure, using survey weights provided by Ipsos to preserve representativeness. For each state, individual exposure indicators were multiplied by their sampling weights, summed across respondents, and divided by the total survey weight in that state. This approach yields the proportion of respondents within each state who reported any cannabis advertising exposure over the past six months. The analytic sample includes respondents from 42 states, and the resulting state-level cannabis advertising exposure (binary measure) ranges from 0.36 to 1.00, reflecting variation in exposure prevalence across states. The resulting state-level exposure variable reflects aggregate exposure environments rather than individual recall and is analytically distinct from external media monitoring data (e.g., Nielsen).

Price-based Sales Promotions (State-level)

In addition to individual-level exposure, we constructed a state-level measure of price-based sales promotion exposure by aggregating weighted individual responses within each state, using the same procedure as for state-level cannabis advertising exposure. This state-level variable reflects the proportion of respondents in each state who reported exposure to any price-based sales promotion over the past six months. The state-level price-based sales promotion exposure ranges from 0 to 0.46 across the 42 states represented in the analytic sample.

Robustness Analyses Using State-level Exposure Measures

Appendix Table A.3 presents models using state-level averages of cannabis advertising exposure and price-based sales promotion rather than individual-level exposure measures. Across both the logistic and split population models, state-level exposure to cannabis advertising is not associated with recent cannabis initiation, and the estimated coefficients are small and statistically insignificant. Similarly, the number of state-level advertising exposure channels shows positive point estimates in some specifications, but these associations are not statistically significant. State-level price-based sales promotion exposure is also not significantly related to recent initiation across models.

II. Tables

Table A.1 Comparison of Sample Characteristics: One-Wave-Only (N=964) vs. Both-Waves (N=532) Respondents

| Samples  Variables | One Wave Only  Mean (SD) | Both Waves  Mean (SD) | Difference | P-value (significance level) |
| --- | --- | --- | --- | --- |
| Exposed to Any Cannabis Advertisement State Average (dum) | 0.73 (0.14) | 0.73 (0.13) | -0.01 | 0.54 |
| Number of Cannabis Advertisement Channels Exposed State Average (con) | 1.65 (0.50) | 1.67 (0.52) | -0.02 | 0.51 |
| Exposed to Any Cannabis Price-based Sales Promotion (dum) | 0.20 (0.12) | 0.20 (0.12) | 0.01 | 0.43 |
| Number of Other Substances | 0.49 (0.89) | 0.49 (0.86) | <0.00 | 0.95 |
| Household Size | 4.07 (1.58) | 4.03 (1.45) | 0.04 | 0.64 |
|  | Frequency (%) | |  |  |
| Cannabis Use Purpose |  |  |  | <0.01 |
| Neither recreational nor medical | 316 (32.78%) | 125 (23.50%) |  |  |
| Medical | 101 (10.48%) | 45 (8.46%) |  |  |
| Recreational | 344 (35.68%) | 253 (47.56%) |  |  |
| Both | 201 (20.85%) | 107 (20.11%) |  |  |
| Don’t Know | 2 (0.21%) | 2 (0.38%) |  |  |
| Age |  |  |  | 0.23 |
| 15 | 148 (15.35%) | 59 (11.09%) |  |  |
| 16 | 156 (16.18%) | 94 (17.67%) |  |  |
| 17 | 114 (11.83%) | 77 (14.47%) |  |  |
| 18 | 173 (17.95%) | 97 (18.23%) |  |  |
| 19 | 189 (19.61%) | 103 (19.36%) |  |  |
| 20 | 184 (19.09%) | 102 (19.17%) |  |  |
| Gender |  |  |  | 0.21 |
| Male | 495 (51.40%) | 290 (54.82%) |  |  |
| Female | 468 (48.60%) | 239 (45.18%) |  |  |
| Race/Ethnicity |  |  |  | 0.67 |
| White, non-Hispanic | 537 (55.71%) | 309 (58.08%) |  |  |
| Black, non-Hispanic | 117 (12.14%) | 71 (13.35%) |  |  |
| Other, non-Hispanic | 35 (3.63%) | 15 (2.82%) |  |  |
| Hispanic | 201 (20.85%) | 100 (18.80%) |  |  |
| 2+, non-Hispanic | 74 (7.68%) | 37 (6.95%) |  |  |
| Education |  |  |  | 0.85 |
| No higher than high school diploma or GED | 698 (72.48%) | 381 (71.62%) |  |  |
| Some college | 235 (24.40%) | 136 (25.56%) |  |  |
| Bachelor's degree or higher | 30 (3.12%) | 15 (2.82%) |  |  |
| Region |  |  |  | 0.69 |
| Northeast | 144 (14.94%) | 89 (16.73%) |  |  |
| Midwest | 228 (23.65%) | 128 (24.06%) |  |  |
| South | 381 (39.52%) | 210 (39.47%) |  |  |
| West | 211 (21.89%) | 105 (19.74%) |  |  |
| Legalization Status |  |  |  | 0.99 |
| Fully Illegal | 87 (9.02%) | 47 (8.83%) |  |  |
| Fully Legal | 451 (46.78%) | 249 (46.80%) |  |  |
| Mixed | 426 (44.19%) | 236 (44.36%) |  |  |

*Note*: “dum” indicates a binary (dummy) variable; “con” indicates a continuous variable. For Use Purpose, “Neither medical nor recreational” includes susceptible non-users.

Table A.2 Comparison of Sample Characteristics: Wave 1 Only (N=478) vs. Both-Waves (N=532) Respondents

| Samples  Variables | Wave 1 Only Mean (SD) | Both Waves Mean (SD) | Difference | P-value (significance level) |
| --- | --- | --- | --- | --- |
| Exposed to Any Cannabis Advertisement State Average (dum) | 0.72 (0.14) | 0.73 (0.13) | -0.01 | 0.23 |
| Number of Cannabis Advertisement Channels Exposed State Average (con) | 1.67 (0.48) | 1.67 (0.52) | <-0.01 | 0.90 |
| Exposed to Any Cannabis Price-based Sales Promotion (dum) | 0.20 (0.11) | 0.20 (0.12) | 0.01 | 0.39 |
| Number of Other Substances | 0.46 (0.90) | 0.49 (0.86) | -0.03 | 0.63 |
| Household Size | 4.02 (1.51) | 4.03 (1.45) | <-0.01 | 0.97 |
|  | Frequency (%) | |  |  |
| Cannabis Use Purpose |  |  |  | <0.01 |
| Neither recreational nor medical | 177 (37.03%) | 125 (23.50%) |  |  |
| Medical | 54 (11.30%) | 45 (8.46%) |  |  |
| Recreational | 161 (33.68%) | 253 (47.56%) |  |  |
| Both | 85 (17.78%) | 107 (20.11%) |  |  |
| Don’t Know | 1 (0.21%) | 2 (0.38%) |  |  |
| Age |  |  |  | 0.45 |
| 15 | 73 (15.27%) | 59 (11.09%) |  |  |
| 16 | 88 (18.41%) | 94 (17.67%) |  |  |
| 17 | 59 (12.34%) | 77 (14.47%) |  |  |
| 18 | 81 (16.95%) | 97 (18.23%) |  |  |
| 19 | 90 (18.83%) | 103 (19.36%) |  |  |
| 20 | 87 (18.20%) | 102 (19.17%) |  |  |
| Gender |  |  |  | 0.89 |
| Male | 260 (54.39%) | 290 (54.82%) |  |  |
| Female | 218 (45.61%) | 239 (45.18%) |  |  |
| Race/Ethnicity |  |  |  | 0.69 |
| White, non-Hispanic | 289 (60.46%) | 309 (58.08%) |  |  |
| Black, non-Hispanic | 44 (9.21%) | 71 (13.35%) |  |  |
| Other, non-Hispanic | 16 (3.35%) | 15 (2.82%) |  |  |
| Hispanic | 88 (18.41%) | 100 (18.80%) |  |  |
| 2+, non-Hispanic | 41 (8.58%) | 37 (6.95%) |  |  |
| Education |  |  |  | 0.89 |
| No higher than high school diploma or GED | 341 (71.34%) | 381 (71.62%) |  |  |
| Some college | 121 (25.31%) | 136 (25.56%) |  |  |
| Bachelor's degree or higher | 16 (3.35%) | 15 (2.82%) |  |  |
| Region |  |  |  | 0.33 |
| Northeast | 75 (15.69%) | 89 (16.73%) |  |  |
| Midwest | 112 (23.43%) | 128 (24.06%) |  |  |
| South | 174 (36.40%) | 210 (39.47%) |  |  |
| West | 117 (24.48%) | 105 (19.74%) |  |  |
| Legalization Status |  |  |  | 0.52 |
| Fully Illegal | 46 (9.62%) | 47 (8.83%) |  |  |
| Fully Legal | 237 (49.58%) | 249 (46.80%) |  |  |
| Mixed | 195 (40.79%) | 236 (44.36%) |  |  |

*Note*: “dum” indicates a binary (dummy) variable; “con” indicates a continuous variable. For Use Purpose, “Neither medical nor recreational” includes susceptible non-users.

 Table A.3 Logistic and Complementary Log-Log Regression Estimates of Cannabis Initiation, State-Level Advertising Exposure (N=138)

|  | Dependent Variable: Cannabis Initiation  (Recent Use) | | | | |
| --- | --- | --- | --- | --- | --- |
|  | Logit Model | | Split Population Model | | |
|  | (1) | (2) | | (3) | (4) |
| **Advertisement and Promotion Exposure** | | | | | |
| Exposed to Any Cannabis Advertisement (State level) | -0.097 |  | | -0.316 |  |
|  | (0.240) |  | | (1.352) |  |
| Number of Cannabis Advertisement Channels Exposed (State level) |  | 0.119 | |  | 0.628 |
|  |  | (0.145) | |  | (0.742) |
| Exposed to Any Cannabis Price-based Sales Promotion (State level) | -0.035 | -0.048 | | -0.187 | -0.238 |
|  | (0.071) | (0.066) | | (0.423) | (0.380) |
| **Demographics** | | | | | |
| Age (ref: 20) |  |  | |  |  |
| 15 | 2.646* | 2.901* | | 2.385* | 2.605** |
|  | (1.146) | (1.177) | | (0.971) | (0.965) |
| 16 | 1.088 | 1.430 | | 1.036 | 1.303 |
|  | (1.095) | (1.115) | | (0.909) | (0.900) |
| 17 | 1.114 | 1.284 | | 1.019 | 1.138 |
|  | (1.217) | (1.232) | | (1.021) | (1.033) |
| 18 | -0.164 | -0.060 | | -0.144 | -0.053 |
|  | (0.886) | (0.889) | | (0.792) | (0.791) |
| 19 | 0.489 | 0.630 | | 0.424 | 0.485 |
|  | (0.705) | (0.714) | | (0.612) | (0.612) |
| Gender (ref: Male) |  |  | |  |  |
| Female | -0.343 | -0.340 | | -0.249 | -0.225 |
|  | (0.483) | (0.484) | | (0.418) | (0.418) |
| Ethnicity (ref: Non-Hispanic) |  |  | |  |  |
| Hispanic | 0.278 | 0.276 | | 0.324 | 0.342 |
|  | (0.625) | (0.628) | | (0.524) | (0.527) |
| Education (ref: High School or below) |  |  | |  |  |
| Some College | 1.331 | 1.472 | | 1.221 | 1.341 |
|  | (0.939) | (0.960) | | (0.767) | (0.769) |
| Bachelor’s Degree or Higher | 0.244 | 0.353 | | 0.346 | 0.455 |
|  | (1.060) | (1.073) | | (0.891) | (0.876) |
| Household Size | -0.184 | -0.098 | | -0.667 | -0.694 |
|  | (0.202) | (0.111) | | (0.698) | (0.712) |
| **Substance Use Behavior and Context** | | | | | |
| Cannabis Use Purpose (ref: Recreational) |  |  | |  |  |
| Neither Medical Nor Recreational | 1.348 | 1.299 | | 1.130 | 1.169 |
|  | (0.988) | (0.992) | | (0.849) | (0.858) |
| Medical | 1.126 | 1.081 | | 0.938 | 0.935 |
|  | (0.785) | (0.787) | | (0.675) | (0.681) |
| Both | 1.183 | 1.168 | | 1.016 | 1.054 |
|  | (0.902) | (0.907) | | (0.765) | (0.783) |
| Number of Other Substances | -0.013 | -0.016 | | -0.091 | -0.098 |
|  | (0.024) | (0.024) | | (0.168) | (0.168) |
| Region (ref: Northeast) |  |  | |  |  |
| Midwest | 0.101 | 0.185 | | -0.114 | 0.022 |
|  | (0.707) | (0.715) | | (0.613) | (0.627) |
| South | 0.748 | 0.796 | | 0.556 | 0.653 |
|  | (0.758) | (0.765) | | (0.624) | (0.640) |
| West | 0.571 | 0.598 | | 0.430 | 0.496 |
|  | (0.792) | (0.804) | | (0.661) | (0.678) |
| Legality (ref: Fully illegal) |  |  | |  |  |
| Fully legal | 0.784 | 0.344 | | 0.512 | 0.212 |
|  | (1.037) | (1.041) | | (0.846) | (0.851) |
| Mixed | -0.211 | -0.310 | | -0.280 | -0.308 |
|  | (0.887) | (0.886) | | (0.740) | (0.737) |
| Constant | -2.480 | -3.854 | | -2.594 | -3.600* |
|  | (2.323) | (2.154) | | (1.971) | (1.788) |
| Observations | 138 | 138 | | 138 | 138 |

*Notes*: Columns (1)–(2) present logistic regression coefficients (log-odds). Columns (3)–(4) present split-population survival model coefficients from complementary log-log specifications. Standard errors are reported in parentheses. Columns (1) and (3) include the binary indicator for any cannabis advertising exposure. Columns (2) and (4) include the categorical measure capturing the number of cannabis advertising channels. * p<0.05, ** p<0.01, *** p<0.001

Table A.4 Logistic and Split-Population Models of Recent Cannabis Initiation Including Only Major and Significant Variables (N=138)

|  | **Dependent Variable: Cannabis Initiation**  **(Recent Use)** | | | |
| --- | --- | --- | --- | --- |
|  | Logit Model | | Split Population Model | |
|  | (1) | (2) | (3) | (4) |
| Exposed to Any Cannabis Advertisement | 1.831* |  | 1.831 |  |
|  | (0.845) |  | (0.966) |  |
| Exposed to Any Cannabis Price-based Sales Promotion | 0.529 | 0.668 | 0.542 | 0.589 |
|  | (0.504) | (0.545) | (0.515) | (0.446) |
| Number of Cannabis Advertisement Channels Exposed (ref: 0) |  |  |  |  |
| 1 |  | 2.088* |  | 1.818* |
|  |  | (0.851) |  | (0.764) |
| 2 |  | 1.213 |  | 1.100 |
|  |  | (0.999) |  | (0.901) |
| ≥3 |  | 1.524 |  | 1.284 |
|  |  | (0.928) |  | (0.829) |
| Age (ref: 20) |  |  |  |  |
| 15 | 2.159** | 1.954* | 2.206 | 1.188* |
|  | (0.800) | (0.813) | (1.224) | (0.502) |
| 16 | 0.416 | 0.434 | 0.379 | -0.031 |
|  | (0.717) | (0.727) | (0.689) | (0.527) |
| 17 | 0.622 | 0.589 | 0.609 | 0.152 |
|  | (0.966) | (0.977) | (0.920) | (0.778) |
| 18 | -0.104 | -0.022 | -0.097 | -0.360 |
|  | (0.805) | (0.813) | (0.769) | (0.638) |
| 19 | 0.561 | 0.629 | 0.529 | - |
|  | (0.634) | (0.643) | (0.603) | - |
| Constant | -3.486*** | -3.410*** | -3.195*** | -2.930*** |
|  | (0.928) | (0.916) | (0.970) | (0.750) |
| Observations | 138 | 138 | 138 | 138 |

*Notes*: Columns (1)–(2) present logistic regression coefficients (log-odds). Columns (3)–(4) present split-population survival model coefficients from complementary log-log specifications. Standard errors are reported in parentheses. Columns (1) and (3) include the binary indicator for any cannabis advertising exposure. Columns (2) and (4) include the categorical measure capturing the number of cannabis advertising channels.

* p<0.05, ** p<0.01, *** p<0.001

Table A.5 Logistic and Split-Population Models of Lifetime Cannabis Initiation Including Only Major and Significant Variables (N=242)

|  | **Dependent Variable: Cannabis Initiation**  **(Lifetime Use)** | | | |
| --- | --- | --- | --- | --- |
|  | Logit Model | | Split Population Model | |
|  | (1) | (2) | (3) | (4) |
| Exposed to Any Cannabis Advertisement | 0.145 |  | 0.175 |  |
|  | (0.439) |  | (0.374) |  |
| Exposed to Any Cannabis Price-based Sales Promotion | 1.220* | 1.279** | 1.009** | 1.076** |
|  | (0.486) | (0.476) | (0.383) | (0.377) |
| Number of Cannabis Advertisement Channels Exposed (ref: 0) |  |  |  |  |
| 1 |  | -0.295 |  | -0.248 |
|  |  | (0.439) |  | (0.369) |
| 2 |  | 0.022 |  | 0.070 |
|  |  | (0.531) |  | (0.444) |
| ≥3 |  | -0.789 |  | -0.539 |
|  |  | (0.851) |  | (0.764) |
| Ethnicity (ref: Non-Hispanic) | | | | |
| Hispanic | 1.244* | 1.212* | 1.127** | 1.075** |
|  | (0.491) | (0.490) | (0.400) | (0.398) |
| Education (ref: High School or below) | | | | |
| Some College | 0.826 | 0.834 | 0.745* | 0.743* |
|  | (0.447) | (0.449) | (0.369) | (0.371) |
| Bachelor’s Degree or Higher | 1.840* | 1.802* | 1.697** | 1.630** |
|  | (0.769) | (0.781) | (0.586) | (0.595) |
| Cannabis Use Purpose (ref: Recreational) |  |  |  |  |
| Neither Medical Nor Recreational | 0.463 | 0.403 | 0.520 | 0.422 |
|  | (0.712) | (0.710) | (0.623) | (0.616) |
| Medical | 1.579*** | 1.522*** | 1.437*** | 1.366*** |
|  | (0.454) | (0.457) | (0.390) | (0.394) |
| Both | 0.292 | 0.199 | 0.334 | 0.232 |
|  | (0.629) | (0.645) | (0.557) | (0.574) |
| Legality (ref: Fully illegal) | | | | |
| Fully legal | -0.737 | -0.788 | -0.533 | -0.557 |
|  | (0.667) | (0.669) | (0.538) | (0.532) |
| Mixed | -1.720* | -1.780** | -1.456** | -1.484** |
|  | (0.673) | (0.678) | (0.539) | (0.540) |
| Region (ref: Northeast) | | | | |
| Midwest | 0.056 | -0.031 | 0.023 | -0.061 |
|  | (0.553) | (0.558) | (0.455) | (0.461) |
| South | -0.268 | -0.297 | -0.358 | -0.381 |
|  | (0.567) | (0.570) | (0.480) | (0.483) |
| West | -2.018** | -2.120** | -1.858** | -1.942** |
|  | (0.781) | (0.797) | (0.668) | (0.680) |
| Constant | -1.743* | -1.352 | -1.957** | -1.600* |
|  | (0.836) | (0.830) | (0.716) | (0.700) |
| Observations | 242 | 242 | 242 | 242 |

*Notes*: Columns (1)–(2) present logistic regression coefficients (log-odds). Columns (3)–(4) present split-population survival model coefficients from complementary log-log specifications. Standard errors are reported in parentheses. Columns (1) and (3) include the binary indicator for any cannabis advertising exposure. Columns (2) and (4) include the categorical measure capturing the number of cannabis advertising channels.

* p<0.05, ** p<0.01, *** p<0.001

Table A.6 Comparison of Sample Characteristics: Recent-use Respondents (N=31) vs. Non-recent-use Respondents (N=107)

| **Variables** | **Observations (Percent)** | | | |
| --- | --- | --- | --- | --- |
|  | Recent Use (N=31) | Non-recent Use (N=107) | Difference | P-value (significance level) |
| **Cannabis Use, Advertisement & Promotion Exposure:** | | | | |
| Exposed to Any Cannabis Advertisement | | | | |
| Yes | 29 (93.55%) | 80 (74.77%) |  | 0.02 |
| No | 2 (6.45%) | 27 (25.23%) |  |  |
| Number of Cannabis Advertisement Channels Exposed (Past 6 Months) | | | | |
| No Exposure | 2 (6.45%) | 27 (25.23%) |  | 0.03 |
| 1 | 15 (48.39%) | 27 (25.23%) |  |  |
| 2 | 4 (12.90%) | 21 (19.63%) |  |  |
| ≥3 | 10 (32.26%) | 32 (29.91%) |  |  |
| Exposed to Any Cannabis Price-based Sales Promotion (Past 6 Months) | | | | |
| Yes | 10 (32.26%) | 19 (17.76%) |  | 0.08 |
| No | 21 (67.74%) | 88 (82.24%) |  |  |
| **Demographics** | | | | |
| Age |  |  |  | 0.15 |
| 15 | 7 (22.58%) | 7 (6.54%) |  |  |
| 16 | 5 (16.13%) | 17 (15.89%) |  |  |
| 17 | 2 (6.45%) | 8 (7.48%) |  |  |
| 18 | 3 (9.68%) | 18 (16.82%) |  |  |
| 19 | 9 (29.03%) | 28 (26.17%) |  |  |
| 20 | 5 (16.13%) | 29 (27.10%) |  |  |
| Gender |  |  |  | 0.43 |
| Female | 12 (38.71%) | 57 (53.27%) |  |  |
| Male | 19 (61.29%) | 50 (46.73%) |  |  |
| Ethnicity |  |  |  | 0.32 |
| Hispanic | 7 (22.58%) | 16 (14.95%) |  |  |
| Non-Hispanic | 24 (77.42%) | 91 (85.05%) |  |  |
| Education |  |  |  | 0.19 |
| High School or below | 11 (35.48%) | 33 (30.84%) |  |  |
| Some College | 14 (45.16%) | 35 (32.71%) |  |  |
| Bachelor’s Degree or Higher | 6 (19.35%) | 39 (36.45%) |  |  |
| **Substance Use Behavior and Context** | | | | |
| Cannabis Use Purpose |  |  |  | 0.41 |
| Recreational | 18 (58.06%) | 55 (51.40%) |  |  |
| Medical | 4 (12.90%) | 11 (10.28%) |  |  |
| Both | 6 (19.35%） | 16 (14.95%) |  |  |
| Neither Medical nor Recreational | 3 (9.68%) | 25 (23.36%) |  |  |
| Region |  |  |  | 0.70 |
| Northeast | 6 (19.35%) | 22 (20.56%) |  |  |
| Midwest | 8 (25.81%) | 28 (26.17%) |  |  |
| South | 10 (32.26%) | 42 (39.25%) |  |  |
| West | 7 (22.58%) | 15 (14.02%) |  |  |
| Legality |  |  |  | 0.25 |
| Fully Legal | 18 (58.06%) | 45 (42.06%) |  |  |
| Fully Illegal | 3 (9.68%) | 10 (9.35%) |  |  |
| Mixed | 10 (32.26%) | 52 (48.60%) |  |  |
| **Mean (SD)** | | | | |
| Household Size | 3.81 (1.38) | 3.95 (1.22) | -0.14 | 0.57 |
| Number of Other Substances | 0.48 (0.57) | 0.56 (0.77) | -0.08 | 0.61 |
| **Observations** | **31** | **107** |  |  |

 Notes: The number of other substances has a range of 0 to 4.

Table A.7 Comparison of Sample Characteristics: Lifetime-use Respondents (N=46) vs. Never-use Respondents (N=196)

| **Variables** | **Observations (Percent)** | | | |
| --- | --- | --- | --- | --- |
|  | Lifetime Use (N=46) | Never Use (N=196) | Difference | P-value (significance level) |
| **Cannabis Use, Advertisement & Promotion Exposure:** | | | | |
| Exposed to Any Cannabis Advertisement | |  |  | 0.46 |
| Yes | 34 (73.91%) | 134 (68.37%) |  |  |
| No | 12 (26.09%) | 62 (31.63%) |  |  |
| Number of Cannabis Advertisement Channels Exposed (Past 6 Months) | | | | |
| No Exposure | 12 (26.09%) | 62 (31.63%) |  | 0.61 |
| 1 | 14 (30.43%) | 68 (34.69%) |  |  |
| 2 | 10 (21.74%) | 29 (14.80%) |  |  |
| ≥3 | 10 (21.74%) | 37 (18.88%) |  |  |
| Exposed to Any Cannabis Price-based Sales Promotion (Past 6 Months) | | | | |
| Yes | 13 (28.26%) | 24 (17.76%) |  | <0.01 |
| No | 33 (71.74%) | 172 (87.76%) |  |  |
| **Demographics** | | | | |
| Age |  |  |  | 0.03 |
| 15 | 5 (10.87%) | 57 (29.08%) |  |  |
| 16 | 13 (28.26%) | 49 (25%) |  |  |
| 17 | 5 (10.87%) | 26 (13.27%) |  |  |
| 18 | 8 (17.39%) | 30 (15.31%) |  |  |
| 19 | 10 (21.74%) | 15 (7.65%) |  |  |
| 20 | 5 (10.87%) | 19 (9.69%) |  |  |
| Gender |  |  |  | <0.01 |
| Female | 23 (50%) | 101 (51.53%) |  |  |
| Male | 20 (43.48%) | 95 (48.47%) |  |  |
| Missing | 3 (6.52%) | 0 (0%) |  |  |
| Ethnicity |  |  |  | 0.13 |
| Hispanic | 13 (28.26%) | 36 (18.37%) |  |  |
| Non-Hispanic | 33 (71.74%) | 160 (81.63%) |  |  |
| Education |  |  |  | <0.01 |
| High School or below | 27 (58.70%) | 145 (74.98%) |  |  |
| Some College | 14 (30.43%) | 47 (23.98%) |  |  |
| Bachelor’s Degree or Higher | 5 (10.87%) | 4 (2.04%) |  |  |
| **Substance Use Behavior and Context** | | | | |
| Cannabis Use Purpose |  |  |  | <0.01 |
| Recreational | 26 (56.52%) | 55 (28.06%) |  |  |
| Medical | 4 (8.70%) | 21 (10.71%) |  |  |
| Both | 6 (13.04%） | 34 (17.35%) |  |  |
| Neither Medical nor Recreational | 10 (21.74%) | 83 (42.35%) |  |  |
| Missing | 0 (0%) | 3 (1.53%) |  |  |
| Region |  |  |  | 0.25 |
| Northeast | 9 (19.57%) | 32 (16.33%) |  |  |
| Midwest | 15 (32.61%) | 47 (23.98%) |  |  |
| South | 18 (39.13%) | 77 (39.29%) |  |  |
| West | 4 (8.70%) | 40 (20.41%) |  |  |
| Legality |  |  |  | 0.15 |
| Fully Legal | 25 (54.35%) | 88 (44.90%) |  |  |
| Fully Illegal | 6 (13.04%) | 15 (7.65%) |  |  |
| Mixed | 15 (32.61%) | 93 (47.45%) |  |  |
| **Mean (SD)** | | | | |
| Household Size | 3.67 (1.37) | 3.88 (1.40) |  | 0.36 |
| Number of Other Substances | 0.48 (0.72) | 0.30 (0.66) |  | 0.10 |
| **Observations** | **46** | **196** |  |  |

 Notes: The number of other substances has a range of 0 to 4.
